# Supplementary material for: Software Application Profile: PHESANT: a tool for performing automated phenome scans in UK Biobank
Source: Int J Epidemiol. 2017 Oct 5;47(1):29–35. doi: 10.1093/ije/dyx204 (PMC5837456; doi:10.1093/ije/dyx204)
Supplement: Supplementary Material [file dyx204_ije-2017-02-0225-file004.docx]

**Software Application Profile: PHESANT: a tool for performing automated phenome scans in UK Biobank**

Louise A C Millard ^1,2a^, Neil M Davies ^1^, Tom R Gaunt ^1^, George Davey Smith ^1^, Kate Tilling ^1^

^1^ MRC Integrative Epidemiology Unit (IEU) at the University of Bristol, University of Bristol, Bristol

^2^ Intelligent Systems Laboratory, Department of Computer Science, University of Bristol, UK

^a^ Email: louise.millard@bristol.ac.uk

SUPPLEMENTARY MATERIAL

**Supplementary section S1: Detailed description of automated processing flow**

This section describes the precise processing flow of each field type, as illustrated in Figure 1 of the main paper. We first describe how we deal with missingness values.

Preprocessing of missingness values

UK Biobank fields may have particular values assigned as missingness (such as “Preferred not to answer” and “Do not know”; see for example field 3786 [http://biobank.ctsu.ox.ac.uk/showcase/field.cgi?id=3786]). For categorical (single) fields, UK Biobank consistently assigns negative values to these, and so we recode negative values to NA for this field type. Negative values of integer and continuous fields are not exclusively reserved for missingness values (for example, field 77 [http://biobank.ctsu.ox.ac.uk/showcase/field.cgi?id=77]), such that we cannot simply recode all negative values to NA, as we do for categorical (single) fields. When integer or continuous fields have missingness values, they also have an assigned data code that describes these missingness values (see for example field 3786 above). Hence, we reassign these missingness values to NA, by specifying these reassignments in the data coding information file. The process for dealing with missingness values in categorical (multiple) fields is a little more involved and is described below.

Continuous field type

Variables with the continuous field type are assigned to either the continuous, ordered categorical or binary data types, as follows. First, we consider the fact that continuous variables may have more than one measurement (for its first measured time point). For instance, spirometry was measured two or three times a few moments apart (see for example field 3062 [http://biobank.ctsu.ox.ac.uk/showcase/field.cgi?id=3062]). When this is the case we take the mean to create a single value per participant.

Next, we check whether fewer than one fifth of participants with a value for this variable have the same value, and if so treat the variable as continuous. We check this because we inverse normal rank transform continuous data type variables, and where a large number of variables have the same value the rank assigned in this transformation is random among these examples, and this would add noise to the data. If more than one fifth of participants have the same value, then we treat this variable as either binary or ordered categorical. If the variable is binary then we assign the binary data type. Otherwise, we discretise this variable into three categories with roughly the same number of participants in each group, ensuring the cut points are between distinct values (see algorithm in Supplementary section S3). After creating this categorical variable we check whether there are at least 10 participants in each of the three categories, and if not then we attempt to treat this variable as binary, by combining either the first and second, or the second and third categories into a single category. If the variable is assigned the binary type we check that more than 10 participants are assigned each value, and if not then this variable is not tested. Examples of these cases are given in Supplementary table 3.

Integer field type

Integer fields are first converted to a single value per participant where there is more than one value (for its first measured time point), by calculating the mean. If there are 20 or more distinct values of this variable they are treated the same as the continuous field type. Otherwise, we remove all values with fewer than 10 participants (and hence remove these participants from the analysis for this variable) and then treat this variable as either binary or ordered categorical data types if there are two, or more than two values, respectively.

Categorical (single) field type

Categorical (single) variables may be assigned to the binary, ordered categorical or unordered categorical data types. We remove categories with fewer than 10 participants. If the variable has only two values it is treated as binary. UK Biobank defines ‘data codes’ to which one or more fields are assigned, and these define the set of categorical values for these fields and their corresponding numeric values. The PHESANT data-coding information file specifies whether a data code of a categorical (single) field defines an ordered or unordered category structure, and we use this information to assign each non-binary categorical (single) field as either an ordered or unordered categorical data type.

*Default values for categorical (single) fields*

Some categorical (single) fields do not explicitly code all values of the field. This is commonly the case for questionnaire fields containing a ‘none’ option, because UK Biobank “did not expect [participants] to select ‘none’ or ‘no’ for every item in grids with lots of options” (see Section 5.3 of the 24-hour diet questionnaire manual [http://biobank.ctsu.ox.ac.uk/showcase/refer.cgi?id=118240]). For example, field 100200 [http://biobank.ctsu.ox.ac.uk/showcase/field.cgi?id=100200] contains responses to the question “How many glasses/cartons/250ml of pure grapefruit juice did you drink yesterday?”, and includes values ranging from half to 6+, but no value for ‘none’. We assume all people who completed the 24-hour recall diet questionnaire (having a value in field 20080 [http://biobank.ctsu.ox.ac.uk/showcase/field.cgi?id=20080]), and who do not have a value in field 100200, have implicitly opted for ‘none’. Default values can be specified for each data code, in the PHESANT data coding information file.

Categorical (multiple) field type

Each categorical (multiple) variable is converted to a set of binary variables, each denoting whether a participant has a given value of this variable. For example, for the variable describing the bread eaten yesterday (field ID=20091; Supplementary figure 2), with values ‘white’, ‘mixed’, ‘wholemeal’, ‘seeded’ and ‘other’, we generate 5 binary variables, white={true,false}, wholemeal={true,false} and so forth.

Categorical (multiple) fields have an added complexity because when a person has no value in this field this may be because: 1) the field values are *incomplete* – they do not contain all possible values (e.g. a participant who does not eat bread cannot choose any option above) or 2) because the data is missing (for example, because they did not respond to a particular question). We deal with this in one of the following three ways, specified in the variable information file:

1) Include participants with a value for field: Where a field is complete we include only participants who selected an option for this field. For example, field 41228 [http://biobank.ctsu.ox.ac.uk/showcase/field.cgi?id=41228] describes who conducted the delivery at a birth, and has options such as ‘hospital doctor’ and ‘midwife’, and also the fields ‘other’ and ‘not known’. Generating a binary variable for the ‘midwife’ value using only those who answered this question contrasts those who had a delivery with a midwife, with those who had a delivery but did not have a midwife.

2) No missingness assumed: In the second case, we assume that there is no missingness across the cohort. This is often appropriate for linked data such as those from hospital records as these should have no missing data and so only have no value if a person has not attended hospital. For example, field 41203 [http://biobank.ctsu.ox.ac.uk/showcase/field.cgi?id=41203] contains disease diagnoses, and so we assume that an absence of an assignment of a particular disease means the participant does not have the disease (rather than that this information is missing).

3) Related field used to determine missingness: In the third case, we use a related data field to determine, for participants with no value for a categorical (multiple) field, whether they are assigned false, or whether their assignment for this field is missing. For example the bread eaten question (field 20091) described above was part of the ‘diet by 24-hour recall’ questionnaire, and so we include all participants who completed this questionnaire (using field 20082 as an indicator of this) in the test for field 20091. Hence, for the ‘white’ binary variable, all those who had white bread yesterday are assigned true and all those who did not check this option but did respond to this questionnaire are assigned false.

*Dealing with categorical (multiple) values denoting missingness*

Some categorical multiple fields include negative numeric values for particular categories denoting missingness (such as “Do not know”). We exclude all participants with a missing value from the *false* value of the generated binary variable, because we cannot know if they do or do not pertain to the *true* value of this binary variable. For example, consider field

41228 [http://biobank.ctsu.ox.ac.uk/showcase/field.cgi?id=41228] describing the type of medical professional who conducted the delivery of a participant’s child, and a participant who has given birth twice and has values “midwife” and “not known” in this field. The generated binary variable for midwife includes this participant in the set of participants corresponding to *midwife=true* because we know that on at least one occasion a midwife conducted the delivery. However, we cannot be certain that a hospital doctor has not conducted a delivery for this participant because the “not known” value could refer to “hospital doctor”. Hence, the generated “hospital doctor” binary variable would not include this participant in the set of participants corresponding to *hospital_doctor=false*, because this is not known.

After generating the binary variables for a categorical (multiple) field we remove those where one of the two values has fewer than 10 participants.

**Supplementary section S2: Dealing with fields with multiple time points or multiple measurements at the same time point**

Fields in UK Biobank may be recorded at several time points, and may also be recorded several times at a single time point. For example, spirometry has (to date) been measured at three time points; the initial assessment visit (2006-2010), the first repeat assessment visit (2012-2013), and the imaging visit. At each of these time points, spirometry was measured two or three times a few moments apart. An example field generated from spirometry is the “forced vital capacity” (field id 3062 [http://biobank.ctsu.ox.ac.uk/showcase/field.cgi?id=3062]). UK Biobank refers to each time point as an “instance”, and the measurements at a single time point as “arrays”. When receiving a UK Biobank dataset the column headings have the format FID_INSTANCE_ARRAY, where FID is the field ID, INSTANCE is the instance number and array is the array index within this instance. For example, column 3062_0_0 would contain the first measurement of field 3062, at the first time point (the initial assessment visit). This naming convention is used in PHESANT to identify the columns that correspond to a field’s first time point, such that these can then be processed together (subsequent time points are not used in this version of PHESANT). For example, when continuous and integer fields have multiple values, at their first measured time point, we calculate the mean to create a single value per participant.

**Supplementary section S3: Algorithm (in pseudocode) for converting continuous variable to ordered categorical variable with three categories**

The following pseudocode shows our algorithm for converting a continuous variable pheno, into an ordered categorical variable with three categories, denoted CAT1, CAT2 and CAT3. The actual R code is given in WAS/equalSizedBins.r in the PHESANT GitHub repository.

**# ideally split points are at CDF=1/3 and CDF=2/3 to give three equal sized categories**

Q1 = quantile(pheno, 1/3)

Q2 = quantile(pheno, 2/3)

minValue <- lowest value in pheno

maxValue <- highest value in pheno

**# edge case where first quantile is lowest value in pheno**

IF Q1==minValue THEN

ASSIGN CAT1 TO ALL WITH pheno==minValue

phenoRemaining <- all in pheno not assigned to CAT1

QQ1 <- quantile(phenoRemaining, 1/2)

minValueRemaining <- lowest value in phenoRemaining

maxValueRemaining <- highest value in phenoRemaining

IF QQ1== minValueRemaining THEN

ASSIGN CAT2 TO ALL WITH phenoRemaining==minValueRemaining

ASSIGN CAT3 TO ALL NOT ASSIGNED CAT1 OR CAT2

ELSE IF QQ1== maxValueRemaining THEN

ASSIGN CAT3 TO ALL WITH phenoRemaining==maxValueRemaining

ASSIGN CAT2 TO ALL NOT ASSIGNED CAT1 OR CAT3

ELSE

ASSIGN CAT2 TO ALL phenoRemaining < minValueRemaining

ASSIGN CAT3 TO ALL phenoRemaining >= minValueRemaining

**# edge case where second quantile is highest value in pheno**

ELSE IF Q2==maxValue THEN

ASSIGN CAT3 TO ALL WITH pheno==maxValue

phenoRemaining <- all in pheno not assigned to CAT3

QQ1 <- quantile(phenoRemaining, 1/2)

minValueRemaining <- lowest value in phenoRemaining

maxValueRemaining <- highest value in phenoRemaining

IF QQ1== minValueRemaining THEN

ASSIGN CAT1 TO ALL WITH phenoRemaining==minValueRemaining

ASSIGN CAT2 TO ALL NOT ASSIGNED CAT1 OR CAT3

ELSE IF QQ1== maxValueRemaining THEN

ASSIGN CAT2 TO ALL WITH phenoRemaining==maxValueRemaining

ASSIGN CAT1 TO ALL NOT ASSIGNED CAT1 OR CAT3

ELSE

ASSIGN CAT1 TO ALL phenoRemaining < minValueRemaining

ASSIGN CAT2 TO ALL phenoRemaining >= minValueRemaining

**# special case where 1/3 and 2/3 quantiles are the same**

ELSE IF Q1 == Q2 THEN

ASSIGN CAT1 TO ALL WITH pheno<Q1

ASSIGN CAT2 TO ALL WITH pheno==Q2

ASSIGN CAT3 TO ALL WITH phenol>Q2

**# main case where values in pheno split nicely into three bins**

ELSE

ASSIGN CAT1 TO ALL WITH pheno<Q1

ASSIGN CAT2 TO ALL WITH pheno>=Q1 AND pheno<Q2

ASSIGN CAT3 TO ALL WITH phenol>=Q2

**Supplementary section S4: Description of UK Biobank participant sample used**

Of the 152 249 participants with genetic data currently available (containing the UK BILEVE samples selected on smoking status), we removed 182 with genetic sex different to reported sex. We removed 31 781 participants of non-European descent. We removed 5323 participants who were identified as being related, having a genetic relatedness greater than 5%, giving a sample of 114 963 participants. A participant flow diagram is given in Supplementary figure 6.SUPPLEMENTARY TABLES

**Supplementary table 1: Genetic variants and weights used to construct BMI genetic score for preliminary MR-pheWAS**

| SNP | Effect allele | Other allele | Effect size | Effect allele frequency |
| --- | --- | --- | --- | --- |
| Novel loci in Table 1 of Locke paper (1) | | | | |
| rs657452 | A | G | 0.023 | 0.399 |
| rs12286929 | G | A | 0.022 | 0.524 |
| rs7903146 | C | T | 0.023 | 0.711 |
| rs10132280 | C | A | 0.023 | 0.699 |
| rs17094222 | C | T | 0.025 | 0.210 |
| rs7599312 | G | A | 0.022 | 0.734 |
| rs2365389 | C | T | 0.020 | 0.581 |
| rs2820292 | C | A | 0.020 | 0.560 |
| rs12885454 | C | A | 0.021 | 0.646 |
| rs16851483 | T | G | 0.048 | 0.067 |
| rs1167827 | G | A | 0.020 | 0.568 |
| rs758747 | T | C | 0.023 | 0.287 |
| rs1928295 | T | C | 0.019 | 0.570 |
| rs9925964 | A | G | 0.019 | 0.647 |
| rs11126666 | A | G | 0.021 | 0.257 |
| rs2650492 | A | G | 0.021 | 0.290 |
| rs6804842 | G | A | 0.019 | 0.571 |
| rs4740619 | T | C | 0.018 | 0.549 |
| rs13191362 | A | G | 0.028 | 0.877 |
| rs3736485 | A | G | 0.018 | 0.468 |
| rs17001654 | G | C | 0.031 | 0.163 |
| rs11191560 | C | T | 0.031 | 0.080 |
| rs1528435 | T | C | 0.018 | 0.623 |
| rs1000940 | G | A | 0.019 | 0.304 |
| rs11583200 | C | T | 0.018 | 0.398 |
| rs9400239 | C | T | 0.019 | 0.696 |
| rs10733682 | A | G | 0.017 | 0.473 |
| rs11688816 | G | A | 0.017 | 0.544 |
| rs11057405 | G | A | 0.031 | 0.898 |
| rs11727676 | T | C | 0.036 | 0.905 |
| rs3849570 | A | C | 0.019 | 0.348 |
| rs6477694 | C | T | 0.017 | 0.357 |
| rs7899106 | G | A | 0.040 | 0.050 |
| rs2176598 | T | C | 0.020 | 0.248 |
| rs2245368 | C | T | 0.032 | 0.173 |
| rs17724992 | A | G | 0.019 | 0.731 |
| rs7243357 | T | G | 0.022 | 0.823 |
| rs2033732 | C | T | 0.019 | 0.747 |
| Novel loci in Table 2 of Locke paper (1) | | | | |
| rs9641123 | C | G | 0.029 | 0.403 |
| rs7164727 | T | C | 0.019 | 0.661 |
| rs492400 | C | T | 0.024 | 0.429 |
| rs2080454 | C | A | 0.017 | 0.390 |
| rs7239883 | G | A | \| 0.023 \| \| --- \| | 0.378 |
| rs2836754 | C | T | 0.017 | 0.628 |
| rs9914578 | G | C | 0.020 | 0.209 |
| rs977747 | T | G | 0.017 | 0.424 |
| rs9374842 | T | C | 0.023 | 0.770 |
| rs4787491 | G | A | 0.022 | 0.535 |
| rs1441264 | A | G | 0.017 | 0.597 |
| rs17203016 | G | A | 0.021 | 0.196 |
| rs16907751 | C | T | 0.047 | 0.903 |
| rs13201877 | G | A | 0.024 | 0.131 |
| rs9540493 | A | G | 0.021 | 0.461 |
| rs1460676 | C | T | 0.021 | 0.158 |
| rs6465468 | T | G | 0.025 | 0.301 |
| rs7715256 | G | T | 0.017 | 0.430 |
| rs6091540 | C | T | 0.030 | 0.702 |
| rs2176040 | A | G | 0.024 | 0.349 |
| Previously known GWAS BMI loci in Extended Data Table 2 of Locke paper (1) | | | | |
| rs1558902 | A | T | 0.082 | 0.397 |
| rs6567160 | C | T | 0.056 | 0.235 |
| rs13021737 | G | A | 0.06 | 0.831 |
| rs10938397 | G | A | 0.04 | 0.430 |
| rs543874 | G | A | 0.048 | 0.206 |
| rs2207139 | G | A | 0.045 | 0.170 |
| rs11030104 | A | G | 0.041 | 0.798 |
| rs3101336 | C | T | 0.033 | 0.605 |
| rs7138803 | A | G | 0.032 | 0.366 |
| rs10182181 | G | A | 0.031 | 0.492 |
| rs3888190 | A | C | 0.031 | 0.394 |
| rs1516725 | C | T | 0.045 | 0.862 |
| rs12446632 | G | A | 0.04 | 0.860 |
| rs2287019 | C | T | 0.036 | 0.819 |
| rs16951275 | T | C | 0.031 | 0.768 |
| rs3817334 | T | C | 0.026 | 0.404 |
| rs2112347 | T | G | 0.026 | 0.633 |
| rs12566985 | G | A | 0.024 | 0.447 |
| rs3810291 | A | G | 0.028 | 0.662 |
| rs7141420 | T | C | 0.024 | 0.519 |
| rs13078960 | G | T | 0.03 | 0.198 |
| rs10968576 | G | A | 0.025 | 0.317 |
| rs17024393 | C | T | 0.066 | 0.026 |
| rs12429545 | A | G | 0.033 | 0.130 |
| rs13107325 | T | C | 0.048 | 0.073 |
| rs11165643 | T | C | 0.022 | 0.585 |
| rs17405819 | T | C | 0.022 | 0.704 |
| rs1016287 | T | C | 0.023 | 0.298 |
| rs4256980 | G | C | 0.021 | 0.648 |
| rs12401738 | A | G | 0.021 | 0.369 |
| rs205262 | G | A | 0.022 | 0.273 |
| rs9581854 (referred to as rs12016871 in Locke paper) | T | C | 0.03 | 0.176 |
| rs12940622 | G | A | 0.018 | 0.561 |
| rs11847697 | T | C | 0.049 | 0.049 |
| rs2075650 | A | G | 0.026 | 0.868 |
| rs2121279 | T | C | 0.025 | 0.122 |
| rs29941 | G | A | 0.018 | 0.673 |
| rs1808579 | C | T | 0.017 | 0.519 |

rs2033529 (weight 0.019) is not used as it is not in the UK Biobank data.

Genetic variants and weights from Locke GWAS meta-analysis (1)

**Supplementary table 2: UK Biobank fields excluded from preliminary MR-pheWAS of BMI**

| **Field IDs** | **Reason excluded from phenome scan** |
| --- | --- |
| 22000, 22001, 22003, 22004, 22005, 22006, 22009, 22010, 22011, 22012, 22013, 22014, 22015, 22050, 22051, 22052 | Genetic data description fields |
| 20012, 20013, 20014, 3059, 3065, 3081, 4268, 4275, 4281, 4287, 5149, 5152, 5155, 5164, 6024, 6074, 6075 | Assessment centre environment (ACE) fields |
| 87, 92 | Polymorphic fields (containing values with mixed data types) |
| 31 | Sex field |
| 34, 52, 21003, 21022 | Age fields |
| 54 | Assessment centre |
| 4232, 4243, 4259, 5090, 5091, 5136, 5138, 5139, 5140, 5141, 5142, 5143, 5144, 5145, 5146, 5147, 5148, 10691 | Categorical (single) field with more than one value |
| 5990, 23207, 23211, 23294, 23303, 41203, 41205, | Not available at time of data download |

**Supplementary table 3: Hypothetical examples demonstrating algorithm to transform continuous fields to ordered categorical (or binary) variables**

|  | Variable value (number of participants with value) | Categories generated, validation and further processing |
| --- | --- | --- |
| Example 1 | Value 0 (N=500)  Value 10 (N=100)  Value 20 (N=3)  Value 30 (N=2)  Value 35 (N=50) | Step 1: Create categorical variable  Category 1 contains value 0 (N=500)  Category 2 contains value 10 (N=100)  Category 3 contains values 20, 30 and 35 (N = 55)  Step 2: Check categories are valid  Categories are valid – all contain at least 10 participants.  *Treat as ordered categorical* |
| Example 2 | Value 0 (N=500)  Value 10 (N=100)  Value 20 (N=3)  Value 30 (N=2) | Step 1: Create categorical variable  Category 1 contains value 0 (N=500)  Category 2 contains value 10 (N=100)  Category 3 contains values 20 and 30 (N = 5)  Step 2: Check categories are valid  Categories are not valid – categories 2 and 3 are merged to create a binary variable.  Step 3: Generate binary variable  Binary variable generated has 500 and 105 participants assigned to each value.  Step 4: Check binary variable is valid  Binary variable is valid, having at least 10 participants assigned to each value.  *Treat as binary* |
| Example 3 | Value 0 (N=500)  Value 10 (N=3)  Value 20 (N=3)  Value 30 (N=2) | Step 1: Create categorical variable  Ordered categorical variable generated with categories:  Category 1 contains value 0 (N=500)  Category 2 contains value 10 (N=3)  Category 3 contains values 20 and 30 (N = 5)  Step 2: Check categories are valid  Categories are not valid as category 3 has less than 10 participants  Step 3: Generate binary variable  Categories 2 and 3 are merged. Binary variable generated has 500 and 8 participants assigned to each value.  Step 4: Check binary variable is valid  Binary variable is not valid because a category is assigned to less than 10 participants.  *Remove from phenome scan* |

Categories created in step 1 are generated using algorithm in Supplementary section S3.SUPPLEMENTARY FIGURES

**Supplementary figure 1: Screenshot of question from UK Biobank Assessment Centre “Touchscreen” questionnaire, asking the participant the question “How would you describe your usual walking pace?” (field ID=924)**


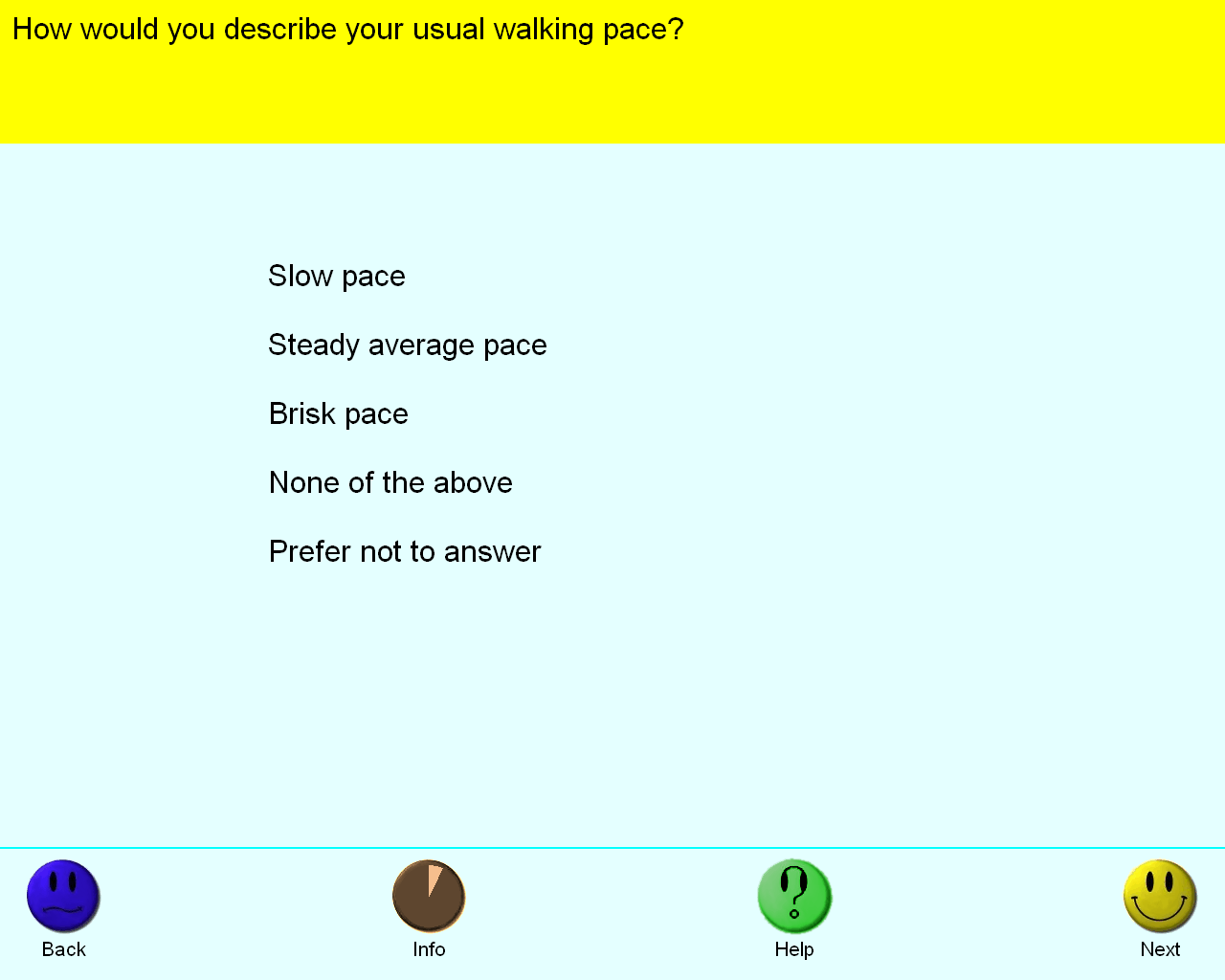


Source: http://biobank.ctsu.ox.ac.uk/showcase/field.cgi?id=924

**Supplementary figure 2: Screenshot of question from UK Biobank Assessment Centre “Diet by 24-hour recall” questionnaire, asking the participant to state the types of bread they ate the day prior to answering the questionnaire (field ID=20091)**

**
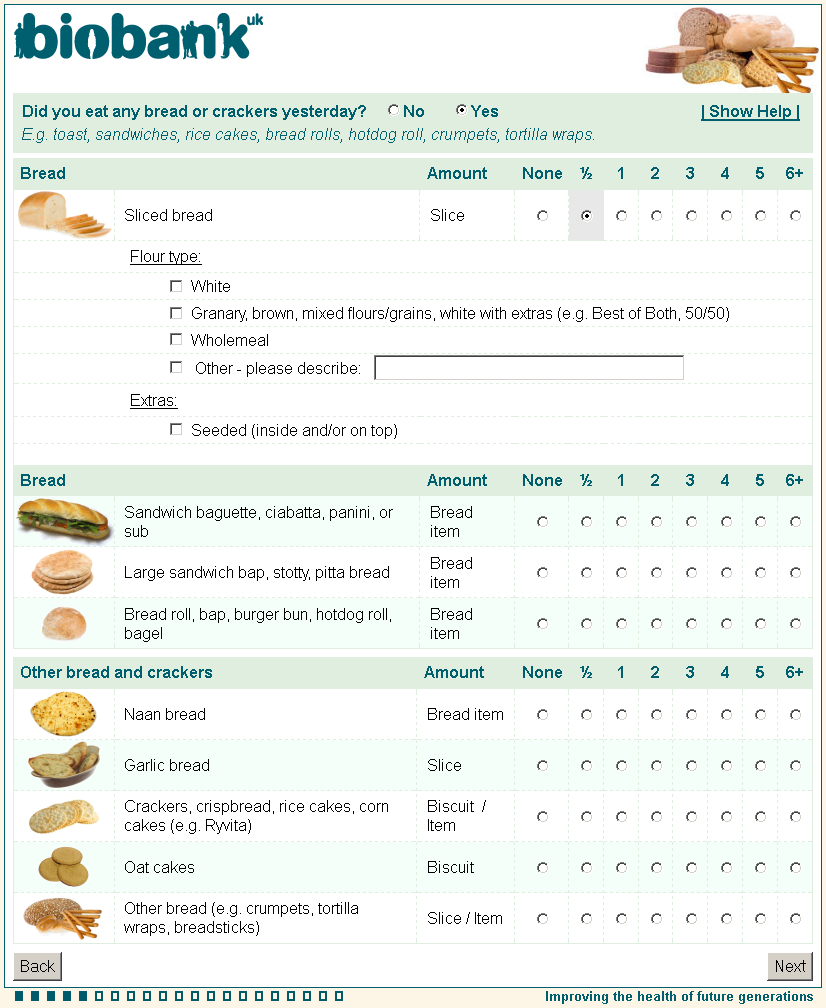
**

Source: http://biobank.ctsu.ox.ac.uk/showcase/field.cgi?id=20091

**Supplementary figure 3: Venn diagrams illustrating options for assignment of value ‘white’=false for generating binary values from field 20091**

Shaded pink area denotes three alternative sets of participants, that can be assigned value ‘white’=false.

**Supplementary figure 4: Variable processing flow diagram showing logic with number of variables reaching each stage of processing flow**

This figure is the same as Figure 1 in the main paper, but with additional red boxes stating the number of variables reaching each stage of the processing flow.

**Supplementary figure 5: Forest plots showing change of phenotype for a 1 standard deviation change of BMI genetic score, for results where P < 4.71x10^-6^ (Bonferroni corrected threshold)**

**A: Variables of the continuous data type**

Standard deviation change of trait of interest for a 1 standard deviation increase of BMI genetic score, and 95% confidence interval.

**B: Variables of the ordered categorical data type**

The change of odds ratio of being in a higher category of trait of interest, for a one standard deviation increase in BMI Genetic score, and 95% confidence interval.

**C: Variables of the binary data type**

Odds ratio of binary trait of interest for a 1 standard deviation increase of BMI genetic score, and 95% confidence interval.

We do not display a forest plot for the multinomial logistic regression results because we use a likelihood ratio test to compare models (the model with the phenotype against the model with confounders only). Hence we do not have an estimate and confidence interval for the whole model overall, with which to generate a forest plot.**Supplementary figure 6: Participant flow diagram**

**References**

1. Locke A, Kahali B, Berndt S, Justice A, Pers TH, Day FR, et al. Genetic studies of body mass index yield new insights for obesity biology. Nature. 2015;518(7538):197–206.
